# *Stenotrophomonas maltophilia* pneumonia in critical COVID-19 patients

**DOI:** 10.1038/s41598-023-28438-x

**Published:** 2023-02-28

**Authors:** Marc Raad, Marc Abou Haidar, Racha Ibrahim, Rouba Rahal, Jocelyne Abou Jaoude, Carine Harmouche, Bassem Habr, Eliane Ayoub, Gebrayel Saliba, Ghassan Sleilaty, Karam Mounzer, Rindala Saliba, Moussa Riachy

**Affiliations:** 1 Pulmonary and Critical Care Department, University Medical Center Hôtel-Dieu de France Hospital, Faculty of Medicine, Saint Joseph University of Beirut, Beirut, Lebanon; 2 Anaesthesia and Critical Care, University Medical Center Hôtel-Dieu de France Hospital, Faculty of Medicine, Saint Joseph University of Beirut, Beirut, Lebanon; 3 Infectious Disease Department, University Medical Center Hôtel-Dieu de France Hospital, Faculty of Medicine, Saint Joseph University of Beirut, Beirut, Lebanon; 4 Cardiovascular Department, University Medical Center Hôtel-Dieu de France Hospital, Faculty of Medicine, Saint Joseph University of Beirut, Beirut, Lebanon; 5 Penn Infectious Disease Penn Presbyterian, Penn Presbyterian Medical Center, Philadelphia, PA USA; 6 Clinical Microbiology Department, University Medical Center Hôtel-Dieu de France Hospital, Faculty of Medicine, Saint Joseph University of Beirut, Beirut, Lebanon

**Keywords:** Microbiology, Infection

## Abstract

*Stenotrophomonas maltophilia*, an environmental aerobic non-fermentative Gram-negative bacilli, has gained attention in many nosocomial outbreaks. COVID-19 patients in intensive care unit have extended hospital stay and are severely immunosuppressed. This study aimed to determine the prevalence and risk factors of *S.* *maltophilia* pneumonia in critical COVID-19 patients. A total of 123 COVID-19 patients in ICU admitted between March 2020 and March 2021 were identified from the authors’ institutional database and assessed for nosocomial pneumonia. Demographic data and factors predisposing to *S. maltophilia* pneumonia were collected and analyzed. The mean age was 66 ± 13 years and 74% were males. Median APACHE and SOFA scores were 13 (IQR = 8–19) and 4 (3–6), respectively. The Median NEWS2 score was 6 (Q1 = 5; Q3 = 8). The Median ICU stay was 12 (Q1 = 7; Q3 = 22) days. *S.* *maltophilia* was found in 16.3% of pneumonia patients, leading to a lengthier hospital stay (34 vs. 20 days; *p* < 0.001). Risk factors for *S.* *maltophilia* pneumonia included previous treatment with meropenem (*p* < 0.01), thrombopenia (*p* = 0.034), endotracheal intubation (*p* < 0.001), foley catheter (*p* = 0.009) and central venous catheter insertion (*p* = 0.016). *S.* *maltophilia* nosocomial pneumonia is frequent in critical COVID-19 patients. Many significant risk factors should be addressed to reduce its prevalence and negative impact on outcomes.

## Introduction

*Stenotrophomonas maltophilia* are Gram-negative bacilli^1^ involved in nosocomial infections, notably hospital-acquired pneumonia (HAP) in the intensive care unit (ICU) setting^2^. *S.* *maltophilia* is known to exhibit intrinsic resistance to several antibiotic classes, including beta-lactams such as carbapenems, as well as aminoglycosides, thus limiting therapeutic options and making *S.* *maltophilia*-related infections hard to manage^3^.

Potential risk factors for acquiring *S. maltophilia* infections were attributed to procedures frequently performed in the ICU^3^, such as arterial and central venous catheters, mechanical ventilation, tracheostomy, Foley catheters, aerosol therapy and the use of active or passive humidifiers^4,5^. Trimethoprim-sulfamethoxazole (TMP-SMX) is the first-line treatment for *S.* *maltophilia* infections, with levofloxacin being the alternative when resistance to trimethoprim-sulfamethoxazole occurs. Other therapeutic options include ceftazidime, ticarcillin-clavulanic acid, tigecycline, and colistin. However, previous exposure to antibiotics could promote the emergence of resistant strains^6^.

Bacterial superinfection occurs in approximately 20% of coronavirus disease of 2019 (COVID-19) patients within two days of intubation and can progress to ventilator-associated pneumonia (VAP)^7^. A recent study showed that COVID-19 patients requiring mechanical ventilation (MV) were significantly more likely to develop VAP than patients requiring MV for other reasons, with an incidence density of 28/1000 ventilator days versus 13/1000 (p-value = 0,009), respectively, thus increasing the mortality rate^8^. A meta-analysis confirmed that around half of mechanically ventilated COVID-19 patients would develop VAP, with high mortality rates^9^. VAP in COVID-19 patients can be polymicrobial, spread to the bloodstream and lead to septic shock^10^.

Data on bacterial superinfection in COVID-19 are scarce or disparate^11^. A limited number of trials have investigated risk factors in ICU patients^4,12^, however, none has focused on *S.* *maltophilia* infections in COVID-19 patients. In fact, there are few publications on the impact of superinfection in patients with COVID-19. To our knowledge, no solid data analyzing the high prevalence of Stenotrophomonas infection were reported in this population apart from isolated case reports^13,14^.

Therefore, this study aimed to determine risk factors for acquiring *S.* *maltophilia* pneumonia in COVID-19 ICU patients, also to compare mortality rates and outcomes between COVID-19 ICU patients with and without superimposed *S.* *maltophilia* infection.

## Materials and methods

### Study design and participants

We conducted a retrospective study at Hôtel-Dieu de France (HDF) university hospital (Beirut, Lebanon) between March 2020 till March 2021, based on the COVID-19 patients’ database. We included all adult patients admitted for at least 48 h to the ICU for COVID-19 infection. Data including patients’ demographic characteristics, clinical history, laboratory results, physiological exams, imaging results, undertaken treatments, and factors predisposing to *S.* *maltophilia* pneumonia were collected. The clinical information was obtained from patient medical charts. The demographic data included age, gender, blood group system ABO and rhesus groups. Comorbidities, such as cardiovascular risk factors, arterial hypertension, diabetes mellitus, immunosuppression, and chronic kidney disease, were also captured. Laboratory exams included : complete blood count, coagulation profile, renal and liver function tests, creatine kinase, lactate dehydrogenase, electrolytes, cardiac enzymes, serum ferritin, C-reactive protein (CRP) and D-dimer. Types of mechanical ventilation and humidification applied were collected.

Stenotrophomonas isolation was performed on quantitative sputum culture in non-intubated patients or on quantitative tracheal aspiration or bronchoalveolar lavage (BAL) in intubated patients.

Data were analysed by physicians from three different departments (pulmonary and critical care, anesthesiology and critical care, and infectious diseases) to resolve any discrepancy in interpretation.

### Ethical considerations

This study was approved and written informed consent was waived in the context of the COVID-19 pandemic by the ethics committee of Hotel-Dieu de France Hospital affiliated to Saint Joseph University (approval number: CEHDF-1630). This study was performed in accordance with the Declaration of Helsinki.

### Microbiology

Respiratory samples, included sputum culture and tracheal aspirates when on mechanical ventilation were collected. They were inoculated, first directly, then after a 100-fold dilution, onto Columbia agar + 5% sheep blood, Chocolate blood agar and 2 additional selective media; MacConkey agar and Columbia agar + 5% sheep blood + Colistin + Nalidixic acid.

*S. maltophilia* species were identified using the BD phoenix automated microbiology system (Becton Dickinson, Sparks, Md., USA). *S.* *maltophilia* species counts were determined after 24 h of growth at 37 °C and expressed as colony-forming unit per milliliter (CFU/mL). Antimicrobial susceptibility testing technique consisted of a determination of the minium inhibitory concentrations (MICs) to TMP-SMX, levofloxacin, ceftazidime and ticarcillin-clavulanic acid, using the same BD phoenix automated system.

### Diagnostic criteria

Sequential Organ Failure Assessment (SOFA)^15^, Acute Physiology and Chronic Health Evaluation II (APACHE II)^16^, and National Early Warning Score (NEWS2)^17^ scores were calculated for each patient upon ICU admission. Invasive procedures during ICU stay, including central venous catheter, urinary catheter, tracheostomy, mechanical ventilation, duration of mechanical ventilation, and duration of ICU stay, were recorded.

The patients were described as acquiring primary SM pneumonia (first pneumonia acquired during ICU stay) or secondary pneumonia (defined as a SM pneumonia acquired after previous pneumonia caused by another pathogen and a recurrence of symptoms and signs after an initial improvement of the patient). Scanographic, baseline laboratory and ICU parameters in the 2 groups without and with Stenotrophomonas infection are summarized in Table 1.

**Table 1:** Scanographic, baseline laboratory and ICU parameters in the 2 groups without and with Stenotrophomonas infection.

	Statistic	Stenotrophomonas		Test	OR (95% CI)	*p* value
		No (103 patients)	Yes (20 patients)			
Scanographic parameters						
@ Baseline						
GGO (%)	m ± sd	30.8 ± 18.6	42.1 ± 23.8	T		0.032
Lobar condensation	N (%)	12 (13.5%)	2 (11.8%)	Chi^2^	0.86 (0.17–4.22)	0.848
Pneumomediastinum	N (%)	1 (1.1%)	0 (0%)	F		0.840
@ 1st Follow-up						
GGO (%)	m ± sd	53 ± 24.3	71.7 ± 22.4	T		0.009
Lobar condensation	N (%)	13 (22.4%)	6 (40%)	Chi^2^	2.31 (0.69–7.69)	0.166
Pneumomediastinum	N (%)	3 (5.2%)	6 (40%)	Chi^2^	12.2 (2.58–57.9)	0.000
Pulmonary embolism	N (%)	7 (12.1%)	0 (0%)	Chi^2^		0.157
Baseline laboratory parameters						
Leucocytes (× 10^3^)	Me [IQR]	8.4 [5.8–11.8]	8.8 [6.4–10.5]	MWU		0.696
Neutrophiles (× 10^3^)	Me [IQR]	6.6 [4.4–10.2]	7.7 [4.9–9.2]	MWU		0.417
Lymphocytes (× 10^3^)	Me [IQR]	.069 [0.48–1.06]	0.73 [0.58–0.82]	MWU		0.869
Lowest platelets level (× 10^3^)	Me [IQR]	141 [100–196]	79 [46–101]	MWU		0.001
Ferritine	Me [IQR]	629 [420–1229]	1239 [360–1739]	MWU		0.280
LDH	Me [IQR]	409 [318–508]	395 [244–707]	MWU		0.867
D-dimers	Me [IQR]	0.89 [0.41–2.01]	0.98 [0.76–3.36]	MWU		0.283
CRP	Me [IQR]	120 [59–186]	147 [95.3–202]	MWU		0.292
Procalcitonine	Me [IQR]	0.26 [0.14–0.56]	0.25 [0.22–0.8]	MWU		0.548
HDL	Me [IQR]	1.01 [0.71–1.25]	1.185 [1.02–1.45]	MWU		0.279
LDL	Me [IQR]	2.67 [1.97–3.35]	2.37 [1.8–3]	MWU		0.593
Triglycerides	Me [IQR]	1.86 [1.30–2.48]	1.96 [1.47–2.20]	MWU		0.976
Serum Creatinin	Me [IQR]	76 [63–111]	94 [79–167]	MWU		0.052
Follow-up ICU parameters						
Highest PEEP	Me [IQR]	8 [3–12]	12 [10–14]	MWU		0.004
Highest FiO^2^	Me [IQR]	1 [0.7–1]	1 [0.8–1]	MWU		0.552
Humid	N (%)	84 (93.3%)	17 (100%)	Chi^2^		0.273
KTA	N (%)	92 (89.3%)	19 (100%)	Chi^2^		0.135
KTC (binary)	N (%)	78 (75.7%)	19 (100%)	Chi^2^		0.016
KTC (quantitative)	Me [IQR]	1 [0–1]	3 [2–3]	MWU		0.000
Number of vascular catheters	Me [IQR]	2 [2–3]	6 [3–6]	MWU		0.000
Foley catheter	N (%)	68 (66%)	19 (95%)	Chi^2^	9.78 (1.26–76.1)	0.009
News 2 Score	Me [IQR]	6 [5–8]	6 [4.5–8]	MWU		0.594
SOFA	Me [IQR]	4 [3–7]	3 [3–5]	MWU		0.162
APACHE	Me [IQR]	13 [8–18]	15 [12–21]	MWU		0.439
ICU transfer day	Me [IQR]	3 [1–6]	3 [1–7]	MWU		0.961
ICU length of stay	Me [IQR]	12 [6–18]	32.5 [20.5–43.5]	MWU		0.000
Intubation day	Me [IQR]	5 [3–10]	8 [3–12]	MWU		0.181
Extubation day	Me [IQR]	13 [11–19]	24 [11–29]	MWU		0.226

Categorical data are presented as frequencies and percentages (N (%)); Continuous data not departing from normality assumptions are presented as mean and standard deviation (m ± sd). Continuous data departing from normality assumptions and ordinal data are presented as Median with its interquartile range (1st quartile–3rd quartile) (Me [IQR]). Chi^2^: Chi square test; MWU: Mann–Whitney U test. For binary data, the odds ratio with its 95% confidence interval were calculated [OR (95% CI)]. F: Fisher exact test. T: independent samples T test.

Treatment received in the last three months before admission and during hospital stay were noted, including agents used for COVID-19 treatment (hydroxychloroquine, azithromycin, ivermectin, remdesivir, lopinavir, glucocorticoids, baricitinib, and tocilizumab) as well as anti-platelets therapy, anti-coagulants therapy, and antibiotics. A high-resolution CT scan was performed on admission day and repeated when indicated. Chest CT severity score (CT-SS) is used to evaluate the severity of pulmonary involvement^18^. Ct-SS was validated using the Cohen’s κ and intraclass correlation coefficient with a strong relationship with the Modified Early Warning Score^19^.

Nosocomial pneumonia was diagnosed using the criteria proposed by the Infectious Diseases Society of America and the American Thoracic Society^20^ and the International guidelines of the ERS/ESICM/ESCMID/ALAT societies^21^. SM-associated pneumonia was diagnosed by a positive microbiologic culture from BAL (> 10^4^ CFU/mL) or tracheal aspirates (> 10^5^ CFU/mL) accompanied by radiographic signs of pulmonary infection (presence of new or increasing infiltrates on chest radiograph) and at least two of the following clinical criteria of pulmonary infection: abnormal temperature, abnormal leucocyte counts or macroscopically purulent tracheal secretions. When confirmed, patients diagnosed with SM pneumonia were treated for 10–14 days with systemic SM/TMP. The control group consisted of all other critically ill patients with COVID-19 but without *S.* *maltophilia* pneumonia. The control group includes patients without pneumonia as well as those with pneumonia due to another pathogen than SM.

### Definition of outcomes

All-cause and ICU mortality, defined as a death event occurring in patients who stayed in the ICU, were recorded. ICU outcomes also included the length of stay, needs for intubation and invasive mechanical ventilation. Cultures and antibiotic sensitivity were analyzed. Dependency outcomes were analyzed using the post-COVID-19 functional status (PCFS)^22^. Safety outcomes were collected and graded according to the “Common Terminology Criteria for Adverse Events” (CTCAE) v5.0^23^. Inflammatory markers were also monitored. The H-score assessed the risk for any reactive hemophagocytic syndrome every 48 h.

### Statistical analyses

Categorical variables were expressed as frequencies and percentages. Continuous variables not departing from normality assumptions (Quartile-Quartile plots, Shapiro–Wilk test) were expressed as mean ± standard deviation, whereas continuous variables with skewed distribution and ordinal variables were expressed as median with interquartile range (1st quartile–3rd quartile). The univariate odds ratio (OR) with its 95% confidence interval (95%CI) was calculated for Boolean categorical variables. Only exposure to potential risk factors before *S.* *maltophilia* acquisition was considered. Chi-squared, Fisher’s exact, and Fisher–Halton–Freeman tests were applied to analyze categorical, as appropriate. The Mann–Whitney U test and the independent-samples T-test were used to compare continuous variables, as appropriate. The analysis was performed on SPSS v26 (IBM Corp. Released 2019. IBM SPSS Statistics for Windows, Version 26.0. Armonk, NY: IBM Corp).

## Results

### Patients’ clinical characteristics

A total of 123 COVID-19 ICU patients (74% men) were enrolled between March 2020 and March 2021, with a mean age of 66.5 ± 13.5 years. The median APACHE score at ICU admission was 13 (Q1 = 8; Q3 = 19), and the SOFA score was 4 (Q1 = 3; Q3 = 6). The median ICU transfer day was three days (Q1 = 1; Q3 = 6), and ICU median duration of stay was 12 days (Q1 = 7; Q3 = 22). Blood type distribution was A group (45.1%), O group (35.3%), B group (12.7%) and AB group (6.9%), with Rhesus positive predominance (92.2%). The most prevalent comorbidities reported were arterial hypertension (67.5%), diabetes mellitus (45.5%), cardiovascular disease (34.1%), renal failure (18.7%) and chronic immunosuppression (8.9%). Protocolized treatment received before ICU admission included systemic steroids (90.2%), ivermectin (77.2%), tocilizumab (32.5%), remdesivir (16.3%), hydroxychloroquine (16.3%), baricitinib (11.4%) and lopinavir/ritonavir (2.4%). The median corticoid dose was equivalent to 20 mg per day of dexamethasone (Q1 = 20; Q3 = 20). The chest CT performed at admission showed important lung involvement with ground-glass opacities (SS-CT 33% ± 20) and lobar consolidation in 13.2% of the patients. Blood exams confirmed high inflammatory markers with a ferritin median of 679 ng/mL (Q1 = 408; Q3 = 1316), LDH of 408 UI/mL (Q1 = 298; Q3 = 515) and lymphopenia of 690 cells/µL (Q1 = 480; Q3 = 970). The median NEWS2 score was 6 (Q1 = 5; Q3 = 8). The oxygen needs on admission in more than 4 l/min and on nonrebreathing mask/Optiflow were 27.6% and 44.7%, respectively.

### Description of SM pneumonia outbreak

**Figure 1:**
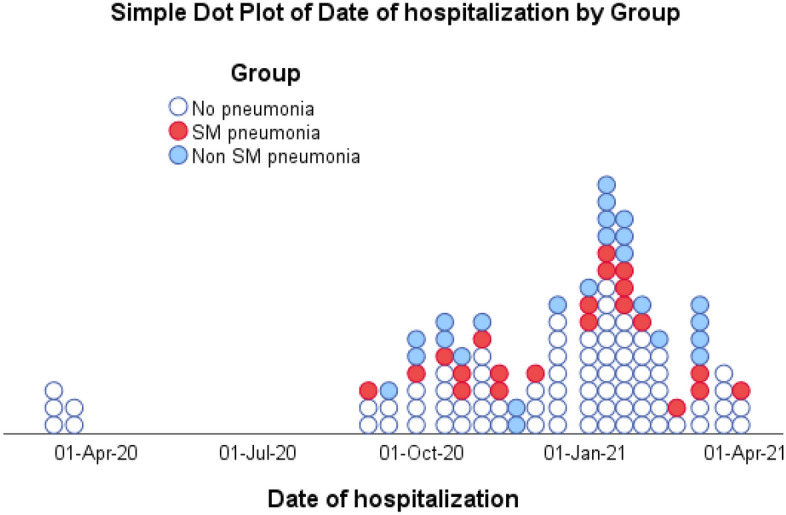
Distribution of critical COVID-19 patients (123 patients) all along the year from March 2020 till March 2021. White dot: Critical COVID-19; Blue dot: non-SM HAP; Red dot: SM HAP.

A total of 42 nosocomial pneumonia events (27.5%) were diagnosed. The most common isolated pathogens were *S.* *maltophilia* in 20 patients (16.2%), *Pseudomonas* spp. and Enterobacterales species in 16 patients (6%) and 4 patients (9.5%), respectively. *Staphylococcus aureus* and *Aspergillus* spp. were rarely reported (2 patients (4.8%) and 4 patients (9.5%), respectively) (Fig. 1). Patients with *S.* *maltophilia* pneumonia were more prone to a second *S.* *maltophilia* pneumonia in 13.8%. Out of the 20 SM pneumonias acquired, 5 were primary infections (25%), while 15 were secondary infections (75%). Demographic data and patient characteristics were divided into two groups, without and with *S.* *maltophilia* infection (Table 2).

**Table 2:** Baseline clinical characteristics, medical history and risk factors in the 2 groups without and with Stenotrophomonas infection.

	Statistic	Stenotrophomonas		Test	OR (95% CI)	*p* value
		No (103 patients)	Yes (20 patients)			
Age	m ± sd	65.5 ± 13.3	68.2 ± 7.8	T		0.384
Male gender	N (%)	75 (72.8%)	16 (80%)	Chi^2^	0.67 (0.21–2.18)	0.503
Blood type						
O	N (%)	33 (38.8%)	3 (17.6%)	Chi^2^		0.015
A	N (%)	39 (45.9%)	7 (41.2%)	Chi^2^		
B	N (%)	10 (11.8%)	3 (17.6%)	Chi^2^		
AB	N (%)	3 (3.5%)	4 (23.5%)	Chi^2^		
Positive Rhesus antigen	N (%)	77 (90.6%)	17 (100%)	Chi^2^		0.188
O2 needs at admission						
No O2	N (%)	10 (9.7%)	1 (5%)	MWU		0.161
O2 < 4 L/min	N (%)	18 (17.5%)	4 (20%)			
O2 4–8 L/min	N (%)	32 (31.1%)	2 (10%)			
MHC/Optiflow	N (%)	42 (40.8%)	13 (65%)			
Endotracheal tube	N (%)	1 (1%)	0 (0%)			
Initial ICU admission	N (%)	51 (49.5%)	9 (45%)	Chi^2^	0.83 (0.32–2.18)	0.712
Day symptoms started	m ± sd	− 6.9 ± 5.8	− 6.1 ± 4.4	T		0.559
1st Ct value	m ± sd	22.8 ± 5.6	20.2 ± 4.4	T		0.113
Weight in kg	m ± sd	84.6 ± 15.3	84.3 ± 18.3	T		0.944
Hypertension	N (%)	71 (68.9%)	12 (60%)	Chi^2^	0.68 (0.25–1.81)	0.435
Diabetes mellitus	N (%)	45 (43.7%)	11 (55%)	Chi^2^	1.58 (0.60–4.13)	0.353
Cardiovascular disease	N (%)	37 (35.9%)	5 (25%)	Chi^2^	0.59 (0.20–1.77)	0.346
Chronic renal failure	N (%)	19 (18.4%)	4 (20%)	Chi^2^	1.11 (0.33–3.68)	0.870
Heart failure	N (%)	14 (14%)	4 (20%)	Chi^2^	1.54 (0.45–5.27)	0.493
Immunosuppression	N (%)	10 (10%)	3 (15%)	Chi^2^	1.59 (0.40–6.38)	0.511
Obstructive lung disease	N (%)	26 (26%)	5 (25%)	Chi^2^	0.95 (0.31–2.87)	0.926
Previous treatment						
Previous pathogen	N (%)	5 (5%)	2 (10%)	Chi^2^	2.11 (0.38–11.74)	0.384
Treatment with meropenem	N (%)	4 (4%)	6 (30%)	Chi^2^	10.39 (2.60–41.5)	0.000
Treatment with quinolone	N (%)	6 (5.9%)	1 (5%)	Chi^2^	0.83 (0.09–7.32)	0.869
Treatment with piperacillin/tazobactam	N (%)	8 (8%)	4 (20%)	Chi^2^	2.88 (0.77–10.7)	0.102

Categorical data are presented as frequencies and percentages (N (%)); Continuous data not departing from normality assumptions are presented as mean and standard deviation (m ± sd). Chi^2^: Chi square test; MWU: Mann–Whitney U test. For binary data, the odds ratio with its 95% confidence interval were calculated [OR (95% CI)]. F: Fisher exact test. T: independent samples T test.

### Risk factors associated with *S. maltophilia* pneumonia

*S. maltophilia* pneumonia was encountered more frequently in group B (17%) and group AB (23.5%) blood types compared to 11.8% and 3.5% in the control group, respectively (*p* = 0.015). Previous antibiotic administration in the last three months was a risk factor for the development of *S.* *maltophilia* pneumonia, especially with meropenem, 30% vs. 4%, respectively, with OR = 10.4 (95% CI 2.6–41.5). Furthermore, antibiotic treatment during the current hospital stay increased this risk, especially carbapenems, 75% vs. 46.6% in the control group, with OR = 3.4 (95% CI 1.16–10.16), and aminoglycosides, 75% vs. 27.2% in the control group, with OR = 8 (95% CI 2.67–24.17) (Table 3). SS-CT with ground-glass opacities in *S.* *maltophilia* pneumonia was 42% ± 24% compared to 31% ± 19% in the control group (*p* = 0.062). On follow-up, *S.* *maltophilia* pneumonia was related to the development of pneumomediastinum in 60% vs. 5.2% in the control group (OR = 12.2; 95% CI 2.58–57.86) and internal hemorrhage in 30% vs. 11.7% in the control group (OR = 3.3; 95% CI 1.05–10.06).

**Table 3:** Treatment characteristics in the 2 groups without and with Stenotrophomonas infection.

	Statistic	Stenotrophomonas		Test	OR (95% CI)	*p* value
		No (103 patients)	Yes (20 patients)			
Hydroxychloroquine	N (%)	18 (17.5%)	2 (10%)	Chi^2^	0.52 (0.11–2.46)	0.407
Azithromycine	N (%)	34 (33%)	6 (30%)	Chi^2^	0.87 (0.31–2.46)	0.793
Ivermectine	N (%)	22 (21.4%)	6 (30%)	Chi^2^	1.58 (0.54–4.58)	0.399
Lopinavir/Ritonavir	N (%)	3 (2.9%)	0 (0%)	F		0.584
Remsidevir	N (%)	17 (16.5%)	3 (15%)	Chi^2^	0.89 (0.24–3.39)	0.867
Glucocorticoids	N (%)	91 (88.3%)	20 (100%)	Chi^2^		0.108
Baricitinib	N (%)	12 (11.7%)	2 (10%)	Chi^2^	0.84 (0.17–4.09)	0.832
Tocilizumab	N (%)	32 (31.1%)	8 (40%)	Chi^2^	1.48 (0.55–3.97)	0.435
IL6 assay results	Me [IQR]	47 [28.7–122.2]	42.2 [24–55]	MWU		0.000
Antibiotics start day	Me [IQR]	1[1–2]	1 [1–1.5]	MWU		0.605
Targeted antibiotics	N (%)	25 (26.3%)	5 (25%)	Chi^2^	0.93 (0.31–2.83)	0.903
Antibiotics association	N (%)	60 (65.2%)	17 (89.5%)	Chi^2^	4.53 (0.98–20.87)	0.053
Penicillin	N (%)	51 (49.5%)	13 (65%)	Chi^2^	1.89 (0.7–5.13)	0.205
Cephalosporin	N (%)	49 (47.6%)	11 (55%)	Chi^2^	1.35 (0.51–3.53)	0.543
Carbapenem	N (%)	48 (46.6%)	15 (75%)	Chi^2^	3.44 (1.16–10.2)	0.020
Aminosid	N (%)	28 (27.2%)	15 (75%)	Chi^2^	8.04 (2.67–24.2)	0.000
Quinolone	N (%)	28 (27.2%)	9 (45%)	Chi^2^	2.19 (0.82–5.85)	0.112
Glycopeptide	N (%)	41 (39.8%)	12 (60%)	Chi^2^	2.27 (0.85–6.03)	0.095
Trimethoprim-sulfamethoxazole	N (%)	7 (6.8%)	14 (70%)	Chi^2^	32 (9.39–109.1)	0.000

Categorical data are presented as frequencies and percentages (N (%)); Continuous data departing from normality assumptions and ordinal data are presented as Median with its interquartile range (1st quartile–3rd quartile) (Me [IQR]). Chi^2^: Chi square test; MWU: Mann–Whitney U test. For binary data, the odds ratio with its 95% confidence interval were calculated [OR (95% CI)]. F: Fisher exact test. T: independent samples T test.

**Figure 2:**
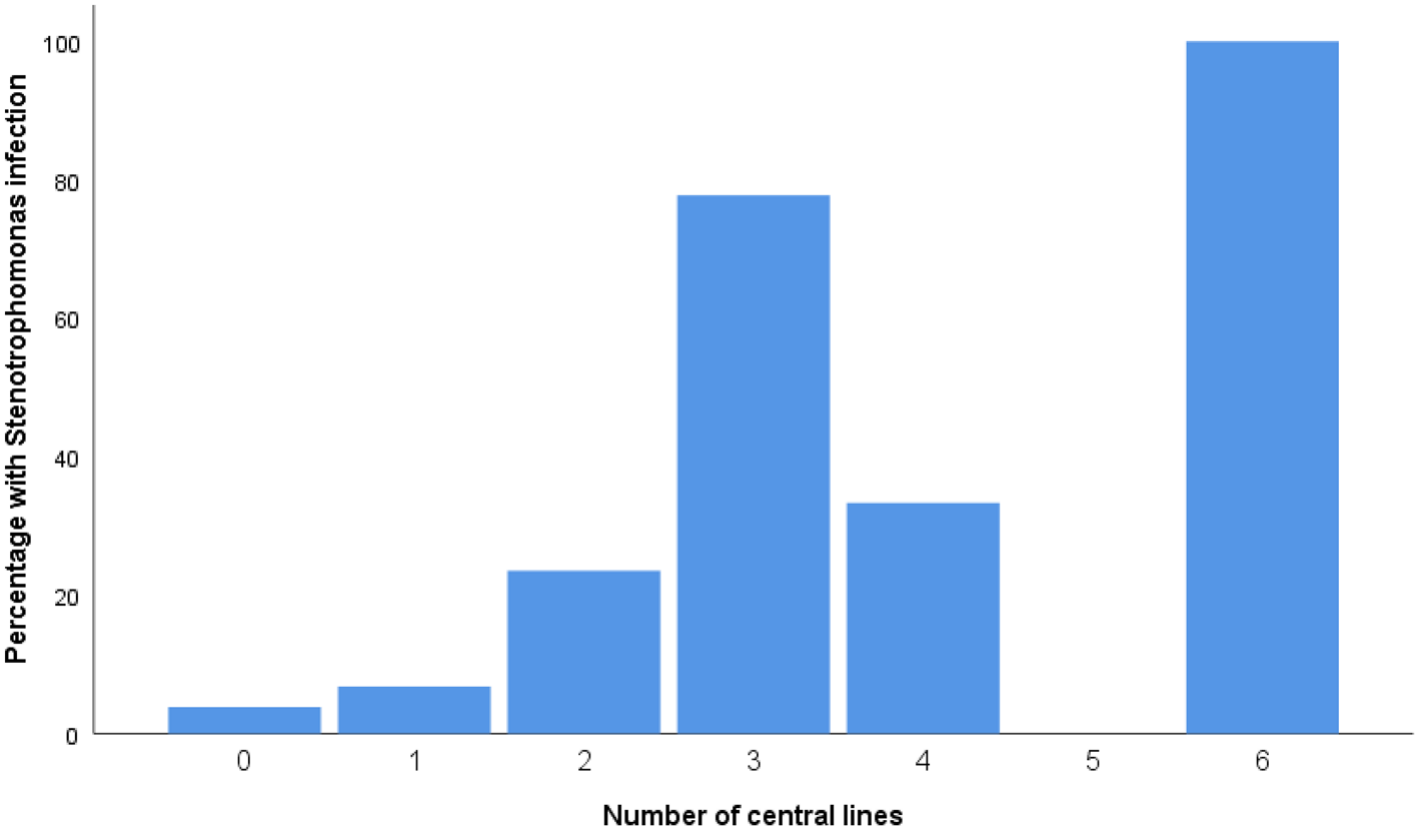
Relation between the probability of acquiring S. maltophilia pneumonia and the number of central lines installed during ICU stay.

Hospital stay in *S.* *maltophilia* pneumonia was 34 days (Q1 = 22; Q3 = 83) compared to 20 days (Q1 = 13; Q3 = 29) in the control group (*p* = 0.0001). ICU stay in *S.* *maltophilia* pneumonia was 33 days (Q1 = 21; Q3 = 44) vs. 12 days (Q1 = 6; Q3 = 18) in the control group (*p* = 0.0001). *S.* *maltophilia* infection showed to be proportional to the number of central lines placed during the ICU stay (*p* = 0.016) (Fig. 2)*.* This finding is also applicable to other installed invasive devices like the urine Foley (OR = 9.8; 95% CI 1.26–76.1) and endotracheal intubation (OR = 16.6; 95% CI 2.14–128.52). On the other hand, factors including the use of active humidifiers or having chronic obstructive pulmonary disease (COPD) were not associated with a higher incidence of SM infection.

### Antimicrobial susceptibility of *S. maltophilia* strains

**Figure 3:**
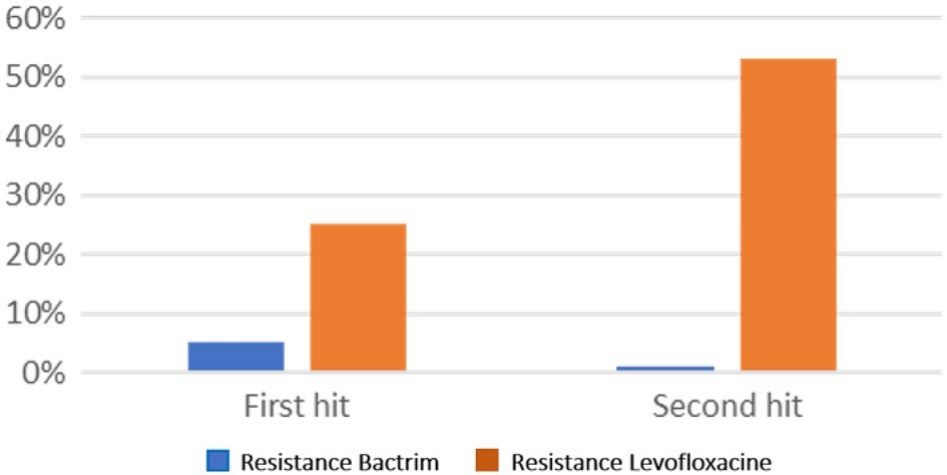
In Vitro resistance of *S.* *maltophilia* pathogen to antibiotics (Trimethoprim-sulfamethoxazole and Levofloxacine) during first and second hit.

In vitro analysis of TMP-SMZ sensitivity was high in the first nosocomial pneumonia (95%) and persisted in the subsequent infection (92.3%). In contrast, levofloxacin sensitivity dropped significantly from 65% at the first infection to 46.2% during subsequent infection (Fig. 3). All *S.* *maltophilia* strains remained sensitive to minocycline and colistin in vitro.

As to the treatment received by the patients, 10 patients received a monotherapy with TMP-SMZ, 6 received a monotherapy with Levofloxacin, while 2 received a combination theraoy with TMP-SMZ and levofloxacine and 2 patients passed away before initiating treatment.

### Outcomes

Clinical and functional outcomes in the 2 groups without and with Stenotrophomonas infection are presented in Table 4. The mortality rate of ICU COVID-19 patients with *S.* *maltophilia* pneumonia was 60% vs. 40% in the control group (p 0.161*).* Lengh of ICU stay for COVID-19 patients with *S.* *maltophilia* pneumonia was prolonged, with a median of 34 days (Q1 = 22; Q3 = 83) vs. 20 days (Q1 = 13; Q3 = 29) (*p* < 0.001).

**Table 4:** Clinical and functional outcomes in the 2 groups without and with Stenotrophomonas infection.

	Statistic	Stenotrophomonas		Test	OR (95% CI)	*p* value
		No (103 patients)	Yes (20 patients)			
Mortality						
N	N (%)	62 (60.2%)	8 (40%)	FFH		0.161
COVID-19-related	N (%)	37 (35.9%)	11 (55%)			
Not COVID-19-related	N (%)	4 (3.9%)	1 (5%)			
Length of stay	Me [IQR]	20 [13–29]	34 [22–82.5]	MWU		0.000
Prone position	N (%)	53 (52.5%)	13 (68.4%)	Chi^2^	1.96 (0.69–5.57)	0.200
Thromboembolic event	N (%)	12 (11.8%)	3 (15%)	Chi^2^	1.32 (0.34–5.19)	0.687
Hemorrhagic event	N (%)	12 (11.7%)	6 (30%)	Chi^2^	3.25 (1.05–10.06)	0.034
Day of Hemorrhagic event	Me [IQR]	17.5 [10.5–21]	20 [14–31]	MWU		0.511
Intubation	N (%)	55 (53.4%)	19 (95%)	Chi^2^	16.6 (2.14–128.5)	0.001
Discharge status						
Autonomous	N (%)	31 (49.2%)	1 (14.3%)	MWU		0.038
O2	N (%)	27 (42.9%)	4 (57.1%)			
Ventilation	N (%)	2 (3.2%)	0 (0%)			
Tracheostomy	N (%)	3 (4.8%)	2 (28.6%)			
PCFS at discharge						
0	N (%)	10 (14.7%)	7 (50%)	MWU		0.242
1	N (%)	7 (10.3%)	0 (0%)			
2	N (%)	19 (27.9%)	1 (7.1%)			
3	N (%)	14 (20.6%)	2 (14.3%)			
4	N (%)	18 (26.5%)	4 (28.6%)			
2-month post-discharge status						
Autonomous	N (%)	47 (81%)	2 (28.6%)	MWU		0.001
O2	N (%)	8 (13.8%)	2 (28.6%)			
Ventilation	N (%)	2 (3.4%)	1 (14.3%)			
Tracheostomy	N (%)	1 (1.7%)	2 (28.6%)			
PCFS @ 2-Month post discharge						
0	N (%)	24 (36.9%)	7 (50%)	MWU		0.710
1	N (%)	24 (36.9%)	1 (7.1%)			
2	N (%)	9 (13.8%)	1 (7.1%)			
3	N (%)	2 (3.1%)	0 (0%)			
4	N (%)	6 (9.2%)	5 (35.7%)			

Categorical data are presented as frequencies and percentages (N (%)); Continuous data departing from normality assumptions and ordinal data are presented as Median with its interquartile range (1st quartile–3rd quartile) (Me [IQR]). Chi^2^: Chi square test; MWU: Mann–Whitney U test; PCFS: Post-COVID-19 Functional Status. For binary data, the odds ratio with its 95% confidence interval were calculated [OR (95% CI)]. FFH: Fisher–Freeman–Halton exact test. T: independent samples T test.

**Figure 4:**
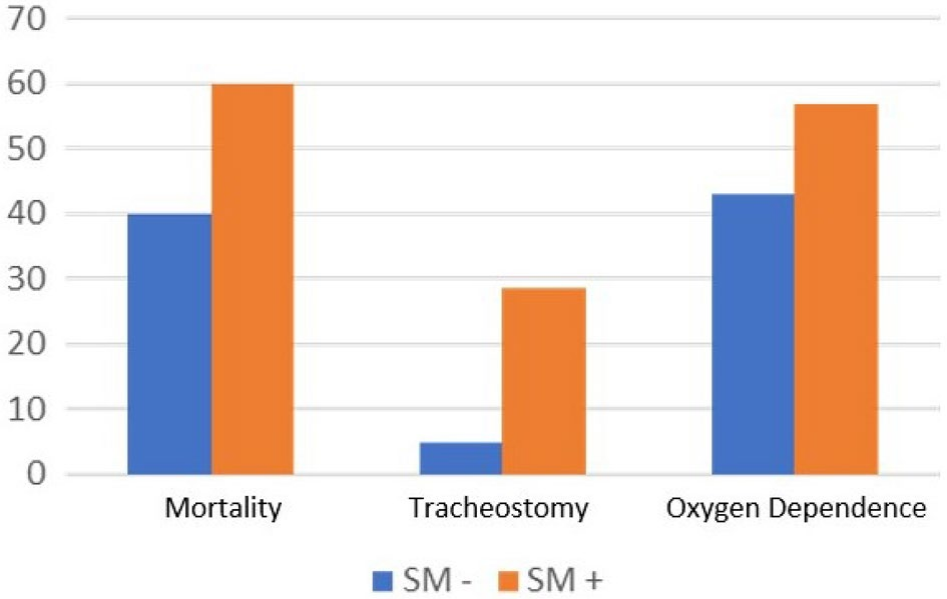
Difference in clinical outcome (in terms of mortality, need for tracheostomy and oxygen dependence) between ICU COVID patients with and without *S.* *maltophilia* superinfection.

Survivors with *S.* *maltophilia* pneumonia had much higher long-term complication rates, i.e., 28.6% tracheostomy, 57% oxygen dependence, and 28.6% ventilation vs. 1.7%, 13.8%, and 3.4%, respectively, in the control group (*p* < 0.001) (Fig. 4).

## Discussion

In this retrospective observational study involving 123 patients with severe COVID-19 infections, 20 (16.3%) were found to have *S. maltophilia*-associated nosocomial pneumonia. The increasing prevalence of *S. maltophilia* pneumonia has been extensively studied. The ubiquitous nature and intrinsic multi-drug resistance of *S.* *maltophilia*, coupled with its ability to rapidly colonize surfaces, such as endotracheal tubes, central venous lines and foley catheters, and form biofilms; a paramount virulence factor rendering it hard to eradicate; contributed to the increasing incidence of nosocomial *S.* *maltophilia* infections, mainly among immunocompromised individuals^1,24^. Surveys from several continents document an increasing isolation rate for *S.* *maltophilia* over the years without reaching rates as high as ours. In England and Wales, the annual number of *S.* *maltophilia* strains isolated from blood samples increased by 93% between 2000 and 2006. Also, a Taiwanese tertiary-care hospital reported an increase of 83% in the prevelance of *S.* *maltophilia* strains recovered from (blood samples?) between 1999 to 2004^1^. However, *S.* *maltophilia*’s prevalence in our study is higher compared to available literature. One multicenter study, conducted among cystic fibrosis patients, showed comparable high rates. The authors found that *S.* *maltophilia* strains have been more frequently isolated from patients’ respiratory samples, noting a considerable variability between centers, with a mean of 4–6% and peaks of 10–25%^25,26^. This significant difference can be attributable to many risk factors that have been linked to the increase of *S.* *maltophilia* pneumonia over the years found in our population, similar to previous findings^27,28^. This high prevalence of *S.* *maltophilia* nosocomial pneumonia observed in our institution can be attributed to known risk factors previously identified that happened to cluster among our patients^27,29^. It is well-established that patients with a severe illness requiring intensive care and taking treatment involving immunosuppressive agents, indwelling devices (e.g., catheters and ventilation tubes), a higher frequency of catheters manipulation, a prolonged and escalating broad-spectrum antibiotic therapy, prolonged intubation, and extended hospital stays are at a greater risk for acquiring *S.* *maltophilia* infections, highly increasing among critically ill populations^27,28^.

Most of these risk factors were present in our sample, explaining the high incidence of *S.* *maltophilia* pneumonia. The development of a biofilm is probably a considerable virulence factor associated with *S.* *maltophilia* infection. It cancolonize surfaces rapidly becoming hard to eradicate^24^. In this study, endotracheal intubation, central venous catheter, urine Foley, and prolonged ICU stay were associated with *S.* *maltophilia* infection. Among immunocompromised COVID-19 patients, *S.* *maltophilia* pneumonia was associated with the presence of a central venous catheter and a Foley catheter. Moreover, the more the catheter was changed, the higher the risk of infection. However, arterial catheters were not associated with a higher risk of infection. Endotracheal tubes are also easily colonized by the pathogen, and infection is associated with a longer duration of intubation and mechanical ventilation^1^. Management of the environment, hospital water supply, and proper handling of medical supplies and equipment play a prominent role in the prevention of *S.* *maltophilia*^1^. Many outbreaks of *S.* *maltophilia* in ICU were reported to emerge from water faucets in contact with respiratory tract and ventilator circuits^30^. In our study, the use of active humidifiers was not associated with more infection than passive humidifiers, and all water faucets specimens in the ICU setting were negative. *S.* *maltophilia* pneumonia occurs in high-risk individuals such as those who had prolonged ICU stays and those who have been on mechanical ventilation for a long time, strongly confirmed by our findings, where extended stays were associated with a higher prevalence of infection. Furthermore, *S.* *maltophilia* infections are a factor for prolonged ICU stay.

In addition to extrinsic risk factors predisposing for *S.* *maltophilia* nosocomial infections, other critical factors intrinsic to the host can impact both the risk of getting infected with *S.* *maltophilia* and outcomes of associated infections. Chronic obstructive pulmonary disease (COPD) has been previously found to be an independent risk factor for ICU-acquired *S.* *maltophilia*^31^. However, COPD did not show to be a significant risk factor in our sample. The proportion of blood group A Rh + in our hospitalized ICU patients with COVID-19 was 45.1%. This was higher than encountered in hospitalized Covid-19 on ward 39.9% (CI 35.2–44.7%), as outpatients 44.8% (CI 39.8–49.9%) in the same period of the pandemic and as controls in the general population 32.3%^32,33^. This is in contrast with the increased risk of *S. maltophilia* pneumonia encountered more frequently in our study in group B and group AB blood types. Other thoracic or systemic complications were found to be significantly related to *S.* *maltophilia* pneumonia. Systemic hemorrhage was an independent significant risk factor for *S.* *maltophilia* secondary infection in 30% of the patients (*p* value < 0.05). To our knowledge, this study is the first that establishes a correlation between acute alveolar hemorrhage and *S.* *maltophilia* infection among COVID-19 patients with no hematologic malignancies or hematopoietic stem cell transplants. This rapid alveolar progressive hemorrhage syndrome associated with *S.* *maltophilia* infection has only been reported in patients with hematologic malignancies^34–37^. Whether the hemorrhage precedes *S.* *maltophilia* infection or the other way around is yet to be determined. It may reflect the severity of the alveolar damage and vascular disruption.

Pneumothorax was another thoracic complication related to *S.* *maltophilia* pneumonia among critically-ill COVID-19 patients^38,39^. In our study, six out of fifteen patients (40%) diagnosed with *S.* *maltophilia* pneumonia had a follow-up CT scan that showed a pneumothorax compared to three out of the 58 patients without *S.* *maltophilia* pneumonia (5.2%). This study is the first to report such a significant association with COVID-19 infected patients, and our literature review only revealed few case reports.

In our study, broad-spectrum antibiotics exposure appeared to be a relevant risk factor for *S.* *maltophilia* pneumonia in ICU admitted patients^40^. Meropenem, aminoglycosides, and TMP-SMX were highly associated with *S.* *maltophilia* infections. Moreover, patients who developed *S.* *maltophilia* pneumonia have been more frequently exposed to antibiotics combination therapy during their ICU stay than the control group (89.5% vs. 65.2%; *p* = 0.037), with higher use of quinolones (45% vs. 27.2%; *p* = ns), carbapenems (75% vs. 46.6%; *p* = 0.02), and aminoglycosides (75% vs. 27.2%; *p* < 0.001).

Trimethoprim-Sulfamethoxazole (TMP-SMZ) and levofloxacin remain the cornerstone of antimicrobial treatment for *S.* *maltophilia* infections^41^. However, the rapid emergence of resistance to these molecules is of concern. They should be initiated once *S.* *maltophilia* has been identified as the cause of nosocomial pneumonia, in order to prevent the risk of colonization, which is often challenging^41^. The molecular mechanisms contributing to its resistance to antibiotics include β-lactamase production, the expression of *Qnr* genes, and the presence of class 1 integrons and efflux pumps^30,42^. Our study showed only 5% (1/20) of TMP-SMX resistance among *S.* *maltophilia* isolated during the first episode of hospital-acquired pneumonia. This rate did not increase during the second episode (1%). In contrast, 25% (5/20) of *S.* *maltophilia* isolated during the first pulmonary infections exhibited resistance to levofloxacin, with an increase to 53.8% (7/13) during the second episode. Previous exposure to fluoroquinolones could have promoted the emergence of resistance to this antibiotic^6^. Furthermore, COVID-19 ICU patients with *S.* *maltophilia* superinfection were more exposed to fluoroquinolones compared to the control group, which can be determinant in the development of acquired resistance. Moxifloxacin is reported to be a little more active than levofloxacin; hence, further exploring its activity could be helpful. Despite colistin susceptibility in vitro, it may not be a very effective drug for many systemic infections, including pneumonia, and would be the last resort drug. While combination therapy has been proposed for *S.* *maltophilia* infections, there is no solid evidence that patients receiving combinations would have better outcomes. Cefiderocol, a fourth-generation siderophore cephalosporin, is usually active in vitro against *S. maltophilia*, including multiresistant isolates. A recent study comparing cefiderocol and high-dose extended-infusion meropenem for nosocomial pneumonia showed non-inferiority between the two drugs, supporting that the drug works well for pneumonia^43^.

As for the clinical outcomes, the mortality varies significantly between COVID-19 patients with and without *S.* *maltophilia* secondary pneumonia. While the median death rate is 40% in ICU COVID-19 patients in the non-*S.* *maltophilia* group, this percentage increases to approximately 60% in those with *S.* *maltophilia*. The survivors have a much higher long-term complication rate at discharge, with a 28.6% tracheostomy rate and 57% oxygen dependence vs. 4.8 and 42%, respectively. This significant difference persists at two months post-discharge. These numbers matches the literature, where overall mortality following *S.* *maltophilia* pneumonia is estimated at 21–69%^27,28,35^. No previous research has been conducted among COVID-19 patients. In our study, it is hard to delineate directly attributable mortality risk factors to *S.* *maltophilia* pneumonia and disease repercussions from those related to the COVID-19 infection itself.

In addition to prompt antibiotic therapy, the implementation of strict measures of infection control, particularly hand hygiene, the use of personal protective equipment, and proper environment cleaning, is critical to reduce the transmission of *S.* *maltophilia-*associated nosocomial infections (including ventilator-associated pneumonia) and limit outbreaks^3–5,44,45^.

If patient-to-patient transmission is suspected, contact isolation precautions for all infected patients on the floor becomes a necessity, in addition to screening stool cultures and/or sputum samples to identify other colonized patients to segregate within the unit^46^. It is recommended to investigate potential infection sources, such as hospital unit water supply, faucets, ice machines, oxygen humidification reservoirs, and any hospital products used mutually between patients (e.g., dialyzer effluent, disinfectant solutions). Reviewing disinfection protocols is warranted to identify any breaches, such as using non-sterile water during these processes.

This study has two merits. First, and to our best knowledge, it is the only study to focus solely on *S.* *maltophilia* infection in COVID-19 patients. While the association of SM with crtitically ill patients is well known, its association with Covid-19 is described for the first time, showing much higher rates than ever seen before. Second, it outlines the most notable risk factor predictors of *S.* *maltophilia* nosocomial pneumonia among critically ill COVID-19 patients, the different antibiotic treatment modalities, infection control measures, and the best approach in investigating potential sources. However, our study has some limitations. Since it was an observational retrospective analysis, some variables that could have impacted the clinical course and disease outcomes could not be controlled at baseline. It is noteworthy to acknowledge the difficulty to differentiate with a high level of certainty whether *S.* *maltophilia* is the culprit agent causing pneumonia or is merely a colonizing bacteria, especially in the context of polymicrobial growth. It was also hard to discern if *S.* *maltophilia* pneumonia is the direct cause of death or just a marker of severe underlying disease mainly driven by comorbidities and complications inherent to a prolonged ICU stay. Thus, death could not be directly linked to *S.* *maltophilia* but was rather classified under hospital mortality. Although this study was conducted in two separate critical care units of a single university hospital in Lebanon, our treatment management, further predisposing risk factors, and antibiotic susceptibility for *S.* *maltophilia* isolates may differ from those in other hospitals. Therefore, our data may not be extrapolated to sites located in other geographic areas. The main limitation of the study remains the fact that it was conducted in a single center. All studied patients were admitted to Hotel Dieu de France hospital’s ICU which may cause a geographic bias. Second, the small number of cases of SM pneumonia encountered prevented the application of multivariate analysis for risk factors and outcomes. Finally, this study was conducted from March 2020 until March 2021, while the vaccine was still not available in Lebanon. All patients included were not yet vaccinated. These results should be confirmed in a vaccinated population.

## Conclusion

*S. maltophilia* nosocomial pneumonia is a worrisome and frequent complication among patients with severe COVID-19 admitted to the ICU and carries significant morbidity and mortality. Identifying patients at high risk for *S.* *maltophilia* nosocomial infection is paramount, as it allows the early recognition and prompt treatment of this infection. The judicious use of antibiotics is crucial, and TMP-SMZ remains the gold standard antimicrobial therapy.

## Data Availability

The datasets generated during and/or analysed during the current study are available from the corresponding author on reasonable request.
